# A Mathematically Generated Noise Technique for Ultrasound Systems

**DOI:** 10.3390/s22249709

**Published:** 2022-12-11

**Authors:** Hojong Choi, Seung-Hyeok Shin

**Affiliations:** 1 Department of Electronic Engineering, Gachon University, Seongnam 13120, Republic of Korea; 2 Department of Mathematics and Big-Data Science, Kumoh National Institute of Technology, Gumi 39177, Republic of Korea

**Keywords:** mathematically generated noise, ultrasound system, mathematical function

## Abstract

Ultrasound systems have been widely used for consultation; however, they are susceptible to cyberattacks. Such ultrasound systems use random bits to protect patient information, which is vital to the stability of information-protecting systems used in ultrasound machines. The stability of the random bit must satisfy its unpredictability. To create a random bit, noise generated in hardware is typically used; however, extracting sufficient noise from systems is challenging when resources are limited. There are various methods for generating noises but most of these studies are based on hardware. Compared with hardware-based methods, software-based methods can be easily accessed by the software developer; therefore, we applied a mathematically generated noise function to generate random bits for ultrasound systems. Herein, we compared the performance of random bits using a newly proposed mathematical function and using the frequency of the central processing unit of the hardware. Random bits are generated using a raw bitmap image measuring 1000 × 663 bytes. The generated random bit analyzes the sampling data in generation time units as time-series data and then verifies the mean, median, and mode. To further apply the random bit in an ultrasound system, the image is randomized by applying exclusive mixing to a 1000 × 663 ultrasound phantom image; subsequently, the comparison and analysis of statistical data processing using hardware noise and the proposed algorithm were provided. The peak signal-to-noise ratio and mean square error of the images are compared to evaluate their quality. As a result of the test, the min entropy estimate (estimated value) was 7.156616/8 bit in the proposed study, which indicated a performance superior to that of GetSystemTime. These results show that the proposed algorithm outperforms the conventional method used in ultrasound systems.

## 1. Introduction

Ultrasound systems can allow physicians to detect diseases for diagnostic purposes [1]; therefore, they can be used to determine the physiological conditions [2]. Ultrasound systems allow physicians to perform immediate treatment in outdoor sports games or island villages [3]. In particular, ultrasound systems have been developed owing to progress in application-specific integrated circuit technology [4,5]; therefore, they have been used to treat patients in island villages [6].

**Figure 1:**
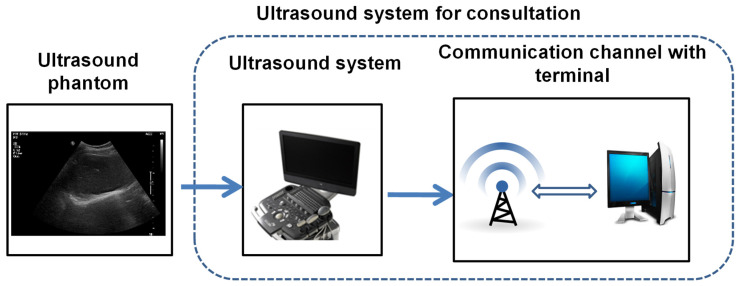
Our proposed concept of an ultrasound system for consultation.

Ultrasound systems comprise access devices and these allow consultation by the physician [7]. Ultrasound is easier to perform without contact; however, ultrasound is currently performed using diagnostic machines, which are susceptible to cyberattacks [8]. Hardware and software development, including encryption algorithms, have been widely applied in automated teller machines and for internet access [9]. Compared with encrypting methods based on hardware, software-based algorithms are easily changeable and can be accessed by a software developer [10]; hence, software-based algorithms for supporting ultrasound have been developed, as shown in Figure 1. In our proposed concept, attacks must be controlled in the terminal machine when necessary.

Our proposed method can be utilized in conventional ultrasound machines since the latter feature 100–240 V alternating-current cords [7,11]; therefore, the encryption algorithm can easily be implemented at the final terminal. Most conventional ultrasound machines are based on outdated Microsoft Windows operating systems [12]. 

Owing to the development of information technology, awareness regarding the importance of information security and encryption technology has increased [13,14]. Encryption technology must satisfy the unpredictable stability of encrypted information protection application systems [15]. Random bits are basic elements in cryptographic algorithm design; therefore, they are vital to the construction of cryptographic systems [16]. These random bits are generated using cryptographic random-bit generators; therefore, they are used to ensure the stability of cryptographic and related application systems. The random-bit generators are classified into pseudorandom-bit generators (PRNGs) and true random-bit generators (TRNGs). 

A cryptographically safe random-bit generator comprises a TRNG and a PRNG so it obtains the output value from the noise source as the input value of the TRNG and uses it as the seed for the PRNG to generate cryptographically safe random bits [17]. Conventional noise sources use noise generated by hardware, such as time, central processing unit (CPU) frequency, hard disk drive revolutions per minute, and memory usage [18]; however, extracting sufficient noise sources from systems is difficult when hardware resources are limited [19]; therefore, we propose a new method to generate a noise source using a mathematical algorithm and verify its performance by comparing it with hardware noise.

Two-post modules in hardware-based number generators were implemented to improve the randomness of linear PRNGs [20]. A chaos-based random number generator in a field-programmable gate array (FPGA) was implemented to achieve higher statistical capability in hardware [21]. Recent random-bit generation techniques for medical imaging applications between years 2018 and 2022 are summarized here. Stochastic gradient descent combined with the random number algorithm was implemented using a private deep learning network for medical imaging applications [22]. A random number was used to generate the weight of a deep learning mechanism for medical imaging [23]. A TRNG-based multilevel fusion technique was developed to improve cryptographic strength for medical imaging applications [24]. A TRNG-based AES encryption algorithm was proposed to develop robust medical systems [25].

Even if power is not supplied in the security field and major information such as hardware chip identification or security key is not lost, the same value should always be maintained when power is supplied again [26,27]; for this purpose, it is mainly stored in a non-volatile memory (NVM) such as an electrically erasable programmable read-only memory [28]; however, in the event of loss or theft of NVM, there is a risk of leakage of important confidential information [29]. It is also exposed to the risk of various physical attacks. One of the ways to solve this problem is the physical uncollectible function (PUF) technology, which generates unpredictable random numbers using process deviations that occur in semiconductor manufacturing processes [30].

The PUF generated from TRNG in the hardware is described. A latency-based dynamic random-access memory PUF without additional error correction technique was utilized for cryptography and authentication service areas [31]. A memory architecture using multi-bit entropy was used for hardware security applications [32]. The combinational TRNG and PUF techniques were designed for resistive random-access memory and FPGA applications [33,34]. The PUF was used for providing a cryptographic key to optimize the elements in the configurable logic block of the FPGA board [35].

Ultrasound is highly susceptible to cyberattacks [36]. In most of the current research, there are various methods for generating noises but most of these studies are based on hardware [37]. Compared with hardware-based encrypting algorithms, software-based algorithms can be updated with software engineers to increase encrypting capability [38]; therefore, we applied a mathematically generated noise function to generate random bits for ultrasound systems.

Section 2 describes the typical noise source generation methods using the CPU of the Microsoft Windows operating system and fundamental information regarding noise sources. Section 3 describes a method to generate random bits using hardware noise and presents the results of random-bit generation and randomized ultrasound images. Section 4 describes a method to derive mathematical functions through a noise generation algorithm for random-bit generation and, then, presents the results of random-bit generation and randomized ultrasound images. Section 5 provides a comparison and an analysis of statistical data processing using hardware noise and the proposed algorithm. Section 6 presents the conclusion and future work of the designed noise generation algorithm.

## 2. Materials and Methods

A method for generating random bits using mathematical functions is proposed herein. To compare and verify this method, hardware noise sources provided by Microsoft’s Windows system were compared with those provided by the proposed algorithm. The hardware noise source depends on the operating environment and operating system, and they tend to exhibit a uniform distribution and low entropy [39]. In this study, the sampling intervals were diversified based on time for the mass generation of random bits. The time-generated time-series data and random-bit generation distributions were extracted and compared with the designed algorithm to evaluate their randomness. In the experiment, a 1000 × 663 bitmap image was randomized with the generated random bit to validate the random bit after randomizing the image.

The random-bit generator generates random bits via the noise-source acquisition, entropy accumulation and seed generation, and random-bit heat generation steps [40]. The output random bit depends on the input noise source which can be evaluated using both hardware and software noise sources [41]. Table 1 shows the noise sources associated with the CPU frequency and CPU elapsed time among the noise sources available in the Microsoft Windows operating system. In the document NIST SP 800-90B, a physical source dependent on hardware and a non-physical noise source using system data are defined [42]; thus, we selected local time and a non-physical noise source to compare performance as a noise source generated by software.

Among the noise sources based on time, GetTickCount and QueryPerformanceCounter use the clock and frequency of the CPU, whereas GetSystemTime uses the time (in microseconds) provided by the system. The GetTickCount, GetSystemTime, and QueryPerformanceCounter from windows API are functions implemented in Microsoft’s C++ language. First, GetTickCount returns the delayed time to the function call in msec from the time the system starts [43]. Second, GetSystemTime returns the current time in Universal Time format [44]. Third, QueryPerformanceCounter returns the current frequency [45]. GetSystemTime uses time-series data and can be created as expressed as shown in Equation (1).

t_n_ = (t_1_ + dt) mod 256, (1)

where t_1_ is the initial time and dt is the amount of change in time.

In Equation (1), mod 256 is a modulo operation that uses 8 bits of the least sequence bit (LSB). A random bit based on time may exhibit higher entropy as the value of dt increases. This implies that random bits require a long time to generate high entropy. QueryPerformanceCounter is a method for measuring the CPU frequency. In Equation (2), frequency is expressed as a random bit based on the initial frequency and elapsed time.

f_n_ = (f_1_ + dt) mod 256, (2)

where f_1_ is the initial frequency and dt is the amount of change in time.

Table 2 lists the frequency counts obtained after the time elapsed by dt. The CPU frequency exhibits a higher rate of change in the 8-bit LSB than in a 24-bit most sequence bit (MSB); therefore, the entropy of the random bit based on the CPU frequency was determined to be 8 bits. As shown in Equation (2), the result of the modulo operation at 256 is combined to generate a random bit of the desired size. The size of the noise source can be generated as 4–64 bytes, but the lower one byte is used as entropy because overlapping values appear in the upper byte.

## 3. Random-Bit Generation by Hardware Noise

**Figure 2:**
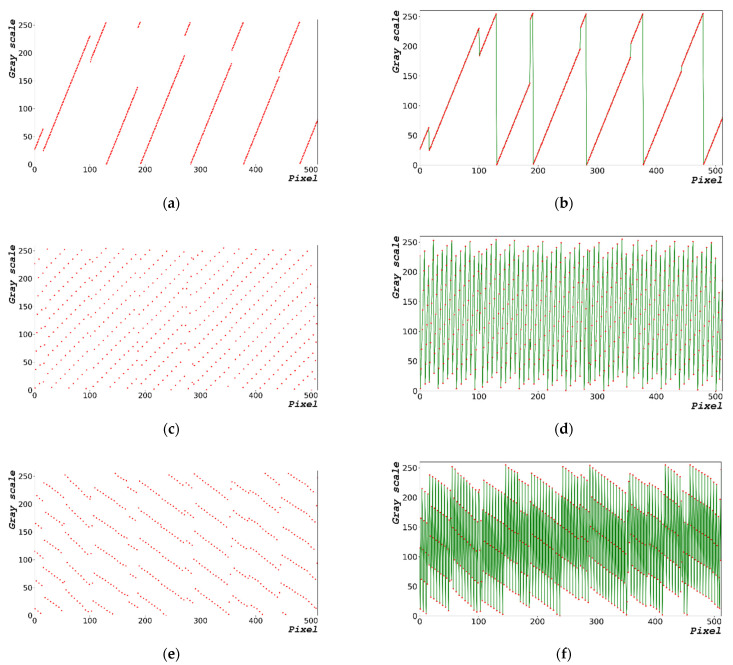
Random-bit generation result based on time: (**a**) 1000 × 663 random-bit generation time-series data; (**b**) generated random-bit trend line for dt = 0 μs; (**c**) 1000 × 663 random-bit generation time-series data; (**d**) generated random-bit trend line for dt = 1.0 μs; (**e**) 1000 × 663 random-bit generation time-series data; and (**f**) generated random-bit trend line for dt = 5.0 μs. Red dot and green line represent the distribution and time flow of the random-bit, respectively.

Figure 2 shows the process of generating random bits with 1000 × 663 pixels. Equation (2) expresses the equation to generate some of the time-series data (0 to 512 pixels) of the random-bit distribution diagram. The pixels of the time-series data on the x-axis represent the flow of time. Figure 2a,c,e show the random-bit generation time-series data at dt = 0, 1.0, and 5.0 μs, respectively. Figure 2b,d,f show the linear trend line representing the distribution and time flow of Figure 2a,e, respectively; in particular, they present the distribution of random-bit generation between 0 and 100 pixels with a linear trend line added. The closer dt is to zero, the higher is the number of random bits that are generated linearly. This implies that the generation of random bits can be predicted. The larger the dt, the greater is the entropy of the random bit; however, the increase in dt significantly increases the overall random-bit generation time.

Table 3 shows the mean, median, and mode of the generated random bit shown in Figure 2. The results are those of a random bit generated in the range of 0 to 255 pixels in a 1000 × 663 image. The generated average was 127 and the median value was 128, which resulted in an even distribution. The trend lines in Figure 2b,d,f show a disadvantage, i.e., the distribution of the generated random bits is linear.

**Figure 3:**
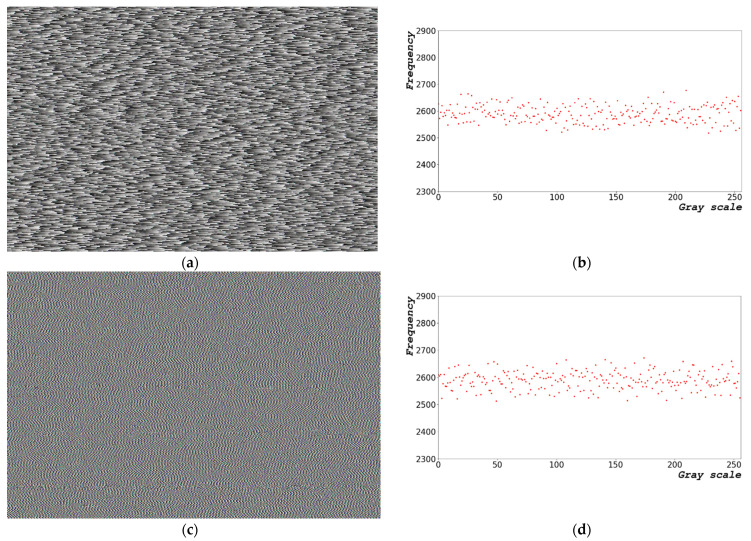

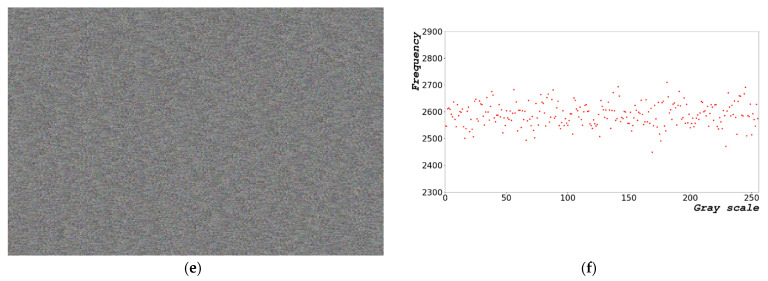
Random-bit generation results based on time: (**a**) 1000 × 663 random-bit visualization image; (**b**) 0–255, 8-bit entropy generation distribution diagram at dt = 0 μs; (**c**) 1000 × 663 random-bit visualization image; (**d**) 0–255, 8-bit entropy generation distribution diagram at dt = 1.0 μs; (**e**) 1000 × 663 random-bit visualization image; and (**f**) 0–255, 8-bit entropy generation distribution diagram at dt = 5.0 μs.

Figure 3 shows the random bits generated using Equation (2). Figure 3a shows an image of the random bit generated and Figure 3b presents the distribution of the 8-bit random bits shown in Figure 3a. The random-bit distribution shaped by the grayscale (Figure 3a) exhibits a constant pattern. In Figure 3a, the overall image form is repeated from dark to bright as it progresses from left to right. Figure 3a,c,e show the 1000 × 663 random-bit visualization images at dt = 0, 1.0, and 5.0 μs, respectively. Figure 3b,d,e show the 0–255, 8-bit entropy generation distribution diagram for dt = 0, 1.0, and 5.0 μs, respectively.

The results shown in Figure 3 indicate that the pattern of random bits generated over time appears as a repeating pattern of constant values from left to right. When the random bit is generated using the frequency counter of the CPU, a certain pattern is yielded, which is disadvantageous.

We attempted to verify the randomness of the noise source by connecting random number candidates of up to 8 bits to generate a noise source of 1024 bits or more. Therefore, we obtain the images using an 8 bit value corresponding to 1 byte so the grayscale range is 0 to 255. The image data of the ultrasound phantom were obtained using an ultrasound machine. 

**Figure 4:**
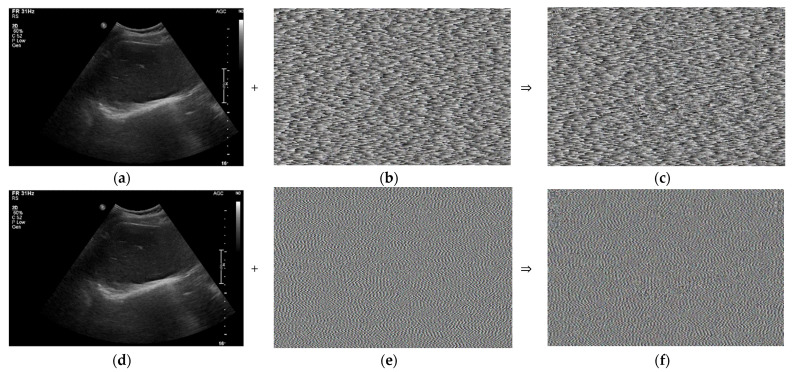

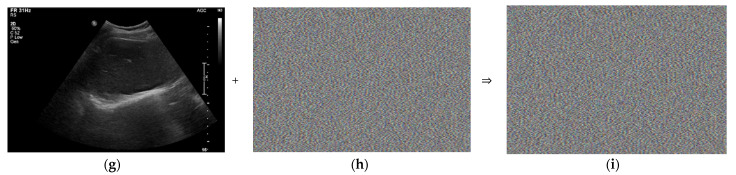
Randomized ultrasound abdomen phantom images: (**a**) ultrasound image; (**b**) 1000 × 663 random-bit generation visualization image; (**c**) randomized ultrasound image at dt = 0.5 μs; (**d**) ultrasound image; (**e**) 1000 × 663 random-bit generation visualization image; (**f**) randomized ultrasound image at dt = 1 μs; (**g**) ultrasound image; (**h**) 1000 × 663 random-bit generation visualization image; and (**i**) randomized ultrasound image at dt = 5.0 μs.

Figure 4a,d,g show the same ultrasound abdomen phantom images captured from the ultrasound machine. Figure 4b,e,h show the 1000 × 663 random-bit generation visualization image at dt = 0, 1.0, and 5.0 μs, respectively. Figure 4c,f,i show the randomized ultrasound images combined from the ultrasound image and random-bit generation visualization image at dt = 0, 1.0, and 5.0 μs, respectively.

## 4. Random-Bit Generation via Designed Noise Generation Algorithm

The random bit is a true random bit if the probability functions Pr for both X and Y variables are not related; thus, we can obtain the following relationship [46]:

Pr (X∪Y) = Pr(X)Pr(Y)(3)

In general, a true random bit cannot be generated or identified; therefore, the random bit is assumed to be deterministic. The deterministic random bit must satisfy a one-to-one corresponding function as shown in Equation (4) [47].

x_k_ ≠ x_k + 1_ ⇔ f(x_k_) ≠ f(x_k + 1_) (4)

where k ∈ N.

**Figure 5:**
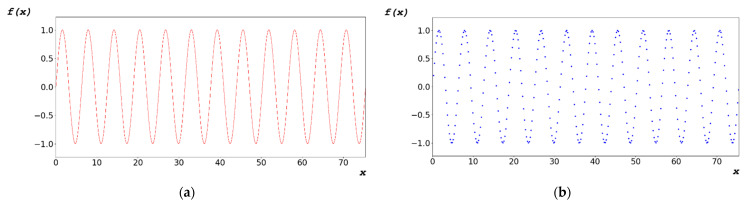
(**a**) Mathematical continuous periodic function; and (**b**) discontinuous periodic function.

As shown in Figure 5a, the periodic function vibrates at regular intervals p, as described in Equation (5). This function does not reflect a one-to-one correspondence, as described in Equation (6).

f(x_k_) = f(x_k_ + p) (5)

x_k_ ≠ x_k + 1_ ⇒ f(x_k_) = f(x_k + 1_) Let x_k + 1_ = x_k_ + p ⇒ f(x_k_) = f(x_k + 1_)⇒ f(x_k_) = f(x_k_ + p) (6)

where k ∈ N and p ∈ R

**Figure 6:**
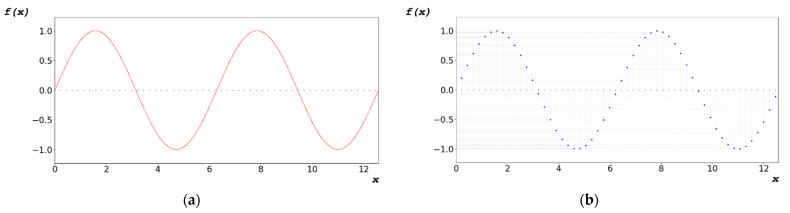
(**a**) Determination of independent variables at regular intervals (dx) for generating random bits in periodic functions; and (**b**) set of non-overlapping dependent variables based on selected independent variables.

To generate the random bit of the algorithm, as shown in Figure 5b, the independent variable x of the domain was generated, as shown in Figure 6a, by adding the change dx of the first term a_1_ and x, as described in Equation (7). The dependent variable y of the function covariate was generated as a random bit, as shown in Figure 6b. The dependent variable y generated using Equation (7) was converted to a floating-point hexadecimal number defined in IEEE754 [48].

a_1_ = current_time( ) x_n_ = a_1_ + ndx, where n ∈ Z y = f(x)(7)

As shown in Figure 6, because the designed algorithm uses a periodic function, an irrational number cannot be used as a period. As described in Equation (8), the rounded value obtained is used at a certain point below the decimal point. The function with a period of rounded q produces an irrational number of q_r_ errors in the normal period, as indicated in Equation (9); therefore, a one-to-one correspondence exists, as in Equation (10), although q is a periodic function.

q = ⌊ p × 10^n^ ⌋ × 10^-n^, where n∈N and p∈I, (8)

q_r_ = p − q (9)

where n is the index, N is a rational number, and I is an irrational number.

The proof for Equation (9) is presented below.

**Theorem** **1.** *Q + I = I*

*Here, Q is a rational number and I is an irrational number.*

**Proof.**  Assume that Q + I = Q

Let a, b, m, and n ∈ Z; subsequently, x ∈ I must be selected.

Furthermore, a, b, m, n, and Z are integer variables.

(10) $$x+\frac{a}{b}=\frac{m}{n}$$

(11) $$x+\frac{a}{b}-\frac{a}{b}=\frac{m}{n}-\frac{a}{b}\Rightarrow \;x\;=\frac{m}{n}-\frac{a}{b}=\frac{\mathrm{mb}-\mathrm{na}}{\mathrm{nb}}$$

(12) $$\Rightarrow \;x=\frac{\mathrm{mb}-\mathrm{na}}{\mathrm{nb}}\;$$

Because variable x is a rational number, this proof is a contradiction; therefore, we confirm that x is an irrational number. □

The designed algorithm satisfies Equation (13) because it uses a periodic function that provides an irrational number. This periodic function is illustrated in Figure 5b.

x_k_ ≠ x_k + 1_ ⇒ f(x_k_) ≠ f(x_k + 1_) f(x) ≠ f(x + q)(13)

**Figure 7:**
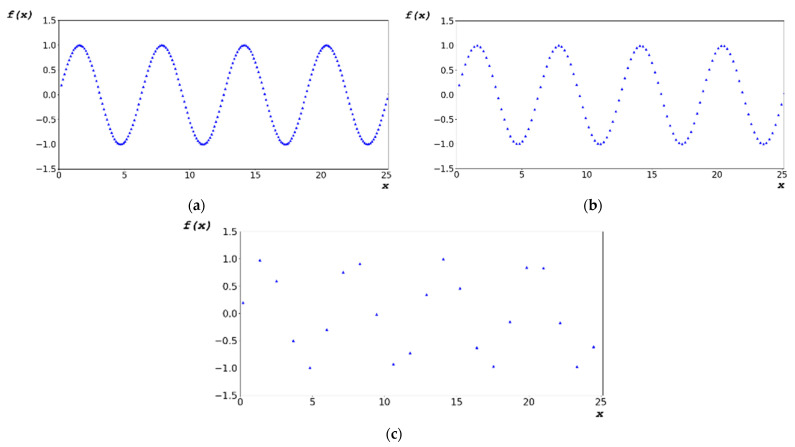
Periodic functions yielded using designed algorithms at (**a**) dx = 0.1156 rad, (**b**) dx = 0.2312 rad, and (**c**) dx = 1.1560 rad.

To test the designed algorithm, a random bit was generated by varying dx, as shown in Figure 7. Figure 7a–c show the periodic functions yielded using the designed algorithms at dx = 0.1156, 0.2312, and 1.1560 rad, respectively.

**Figure 8:**
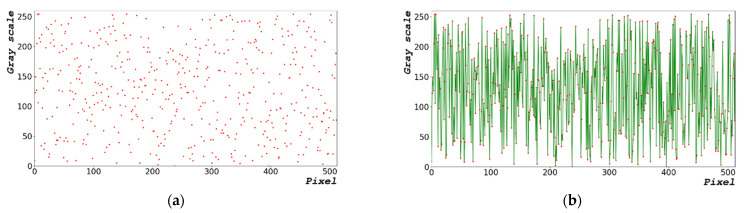

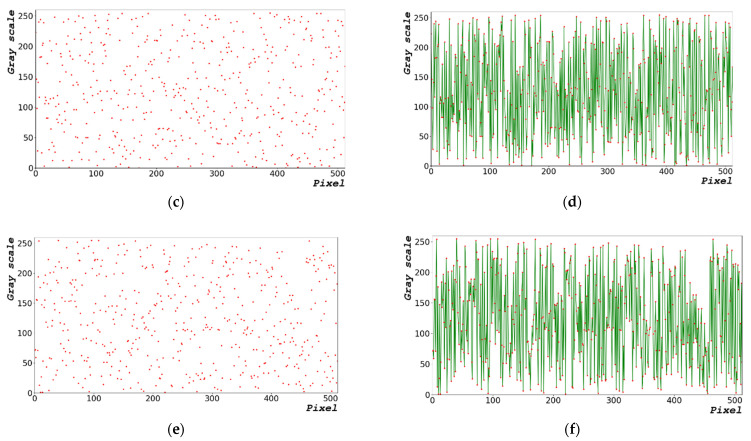
Random-bit generation result in radian: (**a**) 1000 × 663 random-bit generation time-series data; (**b**) trend line of Figure 8a for d_X_ = 0.1156 rad; (**c**) 1000 × 663 random-bit generation time-series data; (**d**) trend line of Figure 8c for dx = 0.2312 rad; (**e**) 1000 × 663 random-bit generation time-series data; and (**f**) trend line of Figure 8c for dx = 1.1560 rad.

Figure 8 shows a graph of the random-bit distribution generated by 1000 × 663 images using the designed algorithm. Figure 8a shows the graph for dx = 0.1156 rad. Figure 8b shows the graph of Figure 8a with a trend line added for d_X_ = 0.1156 rad. Figure 8c,e show the graphs for dx = 0.2312 rad and 1.156 rad, respectively. Based on Figure 2, the data distribution was arbitrarily generated compared with that obtained using the CPU frequency provided by the hardware; in addition, the generation distribution was constant, despite the change in dx.

Table 4 lists the mean, median, and mode of the generated random bits shown in Figure 8. The results are those of a random bit generated in the range of 0 to 255 pixels in a 1000 × 663 image; the generated average was 127, and the median value was 128, which resulted in an even distribution.

**Figure 9:**
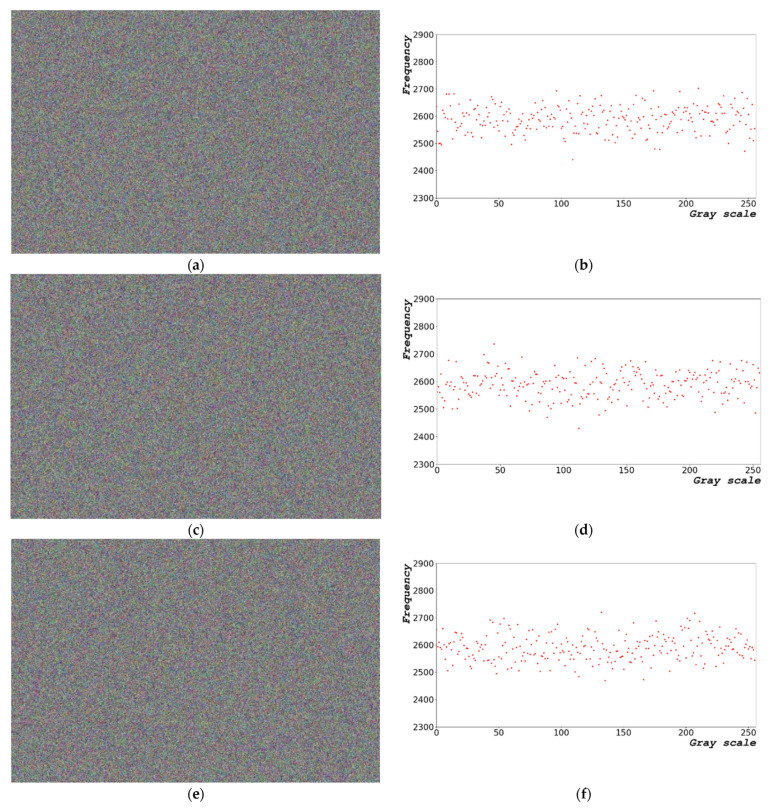
Random-bit generation result based on time: (**a**) image of random-bit generation measuring 1000 × 663; (**b**) distribution diagram chart of entropy generation with size ranging from of 0 to 255 and 8 bits at dx = 0.1156 rad; (**c**) image of random-bit generation measuring 1000 × 663; (**d**) distribution diagram chart of entropy generation with size ranging from 0 to 255 and 8 bits at dx = 0.2312 rad; (**e**) image of random-bit generation measuring 1000 × 663; and (**f**) distribution diagram chart of entropy generation with size ranging from 0 to 255 and 8 bits at dx = 1.1560 rad.

Figure 9a shows an image of the distribution of random bits generated by the designed algorithm. Figure 9b shows the distribution of the generated random bits. Figure 9a,c,e show the images of random-bit generation measuring 1000 × 663 at dx = 0.1156, 0.2312, and 1.1560 rad, respectively. Figure 9b,d,f show the distribution diagrams of entropy generation with sizes ranging from 0 to 255 and 8 bits at dx = 0.1156, 0.2312, and 1.1560 rad, respectively.

**Figure 10:**
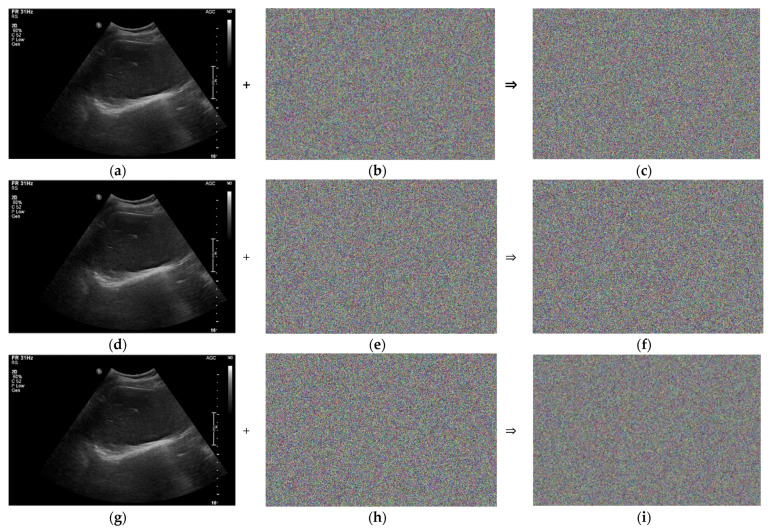
Randomization of abdomen phantom image: (**a**) ultrasound image; (**b**) 1000 × 663 random-bit generation visualization image; (**c**) randomized ultrasound image for dx = 0.1156 rad; (**d**) ultrasound image; (**e**) 1000 × 663 random-bit generation visualization image; (**f**) randomized ultrasound image for dx = 0.2312 rad; (**g**) ultrasound image; (**h**) 1000 × 663 random-bit generation visualization image; and (**i**) randomized ultrasound image for dx = 1.156 rad.

Figure 10a,d,g show the same ultrasound abdomen phantom images. Figure 10b,e,h show the 1000 × 663 random-bit generation visualization image at dx = 0.1156, 0.2312, and 1.1560 rad, respectively. Figure 10c,f,i show the randomized ultrasound images obtained by combining the ultrasound and random-bit generation visualization images at dx = 0.1156, 0.2312, and 1.1560 rad, respectively.

## 5. Comparison Data of Ultrasound Image

**Figure 11:**
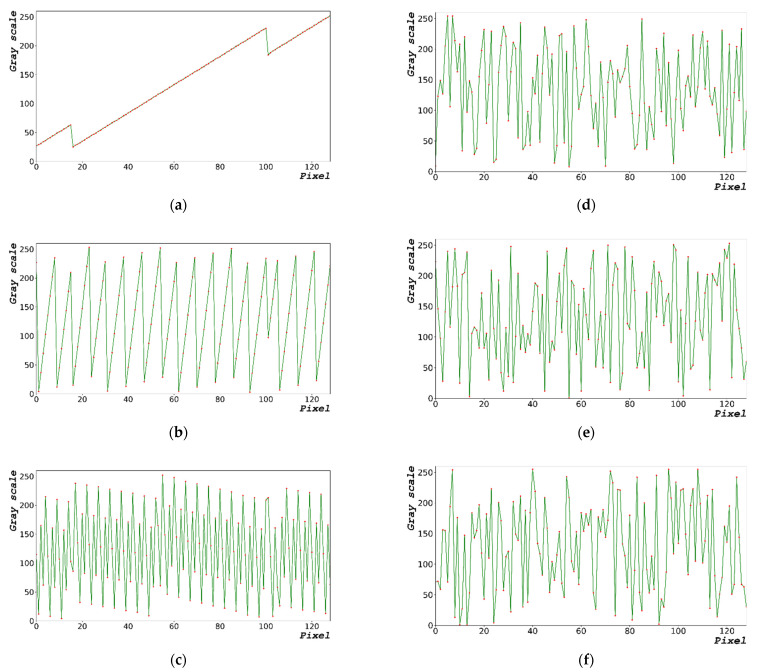
Comparison data for encrypting performance. Enlarged sections of Figures (**a**) 2b, (**b**) 2d, and (**c**) 2f. Enlarged sections of Figures (**d**) 8b, (**e**) 8d, and (**f**) 8f within 0–128 pixels.

Figure 11a–c show the enlarged graphs of Figure 2b,d,f, respectively. Figure 11d–f show the enlarged graphs of Figure 8b,d,f in the range of 0–128 pixels, respectively. The statistical data in Table 3 and Table 4 show only the result; therefore, the distribution of random-bit generation cannot be verified. Based on Figure 11, the random-bit generation process can be analyzed using the random-bit generation trend line and distribution. Compared with the randomized data shown in Figure 11a–c, the randomized data as shown in Figure 11d–f are more complex.

**Figure 12:**
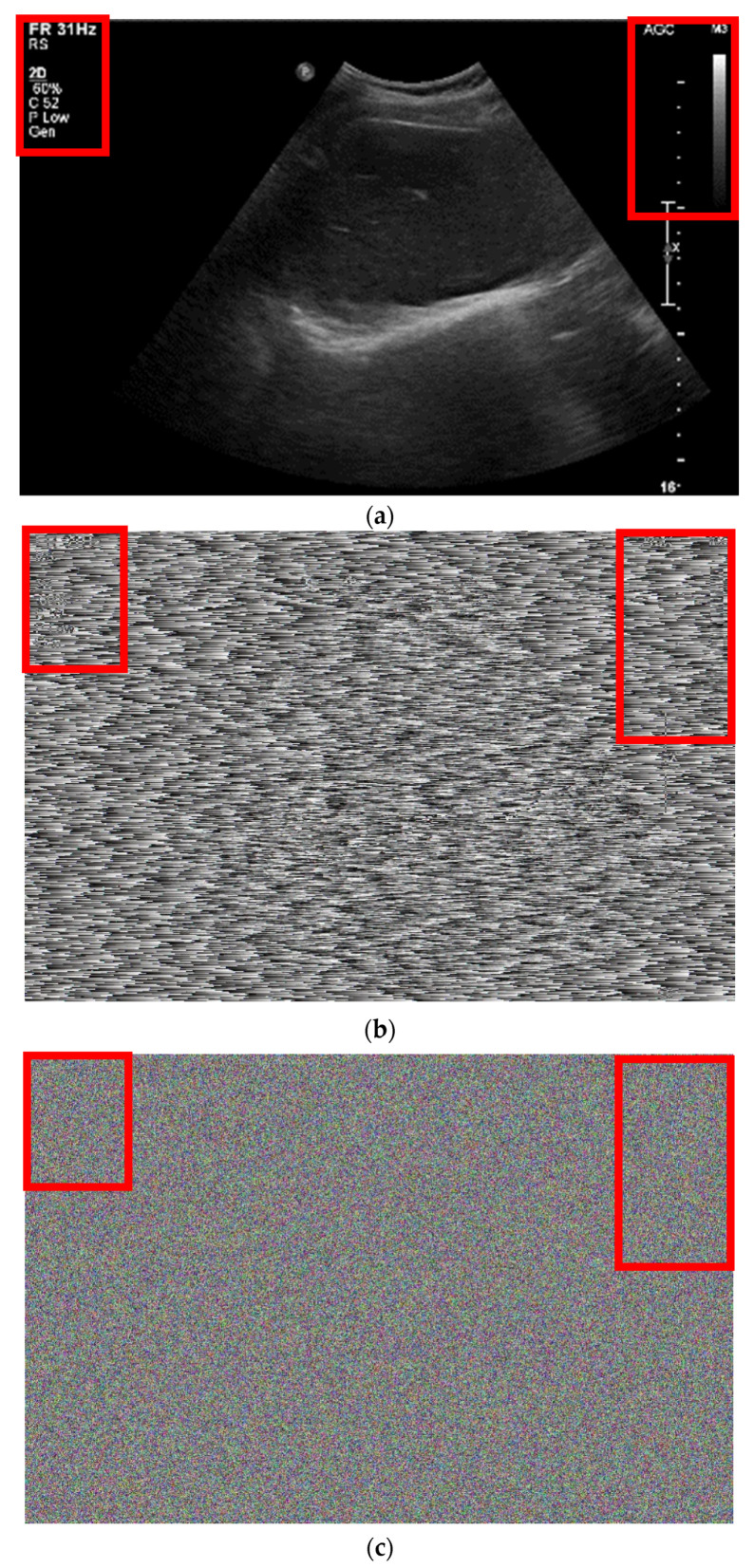
(**a**) Obtained original ultrasound image and enlarged images of (**b**) Figure 4i and (**c**) Figure 10i.

Figure 12b,c show the enlarged images of Figure 4i and Figure 10i, respectively. Figure 11b shows the randomization by the conventional hardware noise source, which allows the original image pattern to be maintained. Figure 12c shows the randomization by the designed algorithm, which does not maintain the pattern of the original image.

Table 5 lists the noise measurement results of the randomized images in Figure 11b,c based on the original image shown in Figure 11a. The mean square error (MSE) is a value representing the error between the reference and comparison images [49]. The peak signal-to-noise ratio (PSNR) is calculated using Equation (14) [50].

(14) $$\mathrm{PSNR}=10\log\;\frac{s^{2}}{\mathrm{MSE}}\;,$$

where s is the maximum value of the pixel.

Table 5 shows the comparison data of the image quality based on the conventional method using hardware noise and based on the proposed method using the designed algorithm. In the conventional method, random bits are generated by the hardware noise. In the proposed method, random bits are generated by software. The MSE is the difference in quality between the original and comparison images [51]; therefore, a high MSE value indicates a significant difference between the original and comparison images. As shown in Equation (14), the PSNR is a metric for evaluating an image and is inversely proportional to the MSE.

Table 5 shows the noisy images based on hardware noise sources (conventional) shown in Figure 4c,f,i, and those based on the proposed algorithms shown in Figure 10c,f,i (proposed). The MSE and PSNR values shown in Table 5 are extracted from Figure 4c,f,i, and Figure 10c,f,i, respectively. The MSE of the proposed algorithm is higher than that yielded by the method using hardware noise.

Table 6 shows the test result of the min entropy estimate (estimated value) vs. time. As a result of the test, the min entropy estimate (estimated value) was 7.156616/8 bit in the proposed study, which showed superior performance than GetSystemTime (Conventional).

## 6. Conclusions

Ultrasound systems have been widely used owing to their applicability. Ultrasound systems that can provide consultation are more likely to be hacked via cyberattacks; therefore, hardware- or software-based algorithms have been implemented to protect confidential patient information. Compared with hardware-based algorithms, software-based algorithms can be easily accessed by software engineers. In our proposed concept for ultrasound systems, an encryption algorithm is implemented in ultrasound machines via a terminal. Such ultrasound systems necessitate random bits to protect patient information. Unpredictable noise generated in the hardware is typically used to generate a random bit; however, extracting sufficient noise from systems is challenging when hardware resources are limited.

Herein, we proposed a new random-bit generation algorithm that can be used in ultrasound systems. Mathematically generated random bits are limited by the hardware memory size; however, the proposed algorithm is not constrained by the memory size because it uses values that diverge vibrations within a certain range using periodic functions. It can generate noise using a mathematical algorithm in a hardware-constrained system, such as an ultrasound system; subsequently, we verified its randomness performance via several experiments. 

To confirm the feasibility of our proposed method, we extracted the images of an ultrasound phantom from an ultrasound system and then compared the image quality using the conventional and proposed methods. The MSE obtained when using the proposed algorithm (17,953.607) was higher than that when using hardware noise (17,933.858), and the PSNR when using the proposed algorithm (5.589) was lower than that when using hardware noise (5.594).; therefore, the randomized image obtained using the proposed algorithm indicated further deterioration in the image quality, which shows that the entropy for the random bit of the proposed algorithm is greater than that of the method that uses hardware noise for the ultrasound system. Our proposed method showed noise generation technique. In the future work, the developed technique will be applied to the embedded system and, then, generate actual key generation and use it as an entropy to increase encryption strength; in addition, research on efficiency in an actual system using the developed technique will be conducted.

## Figures and Tables

**Table 1:** Noise source associated with the CPU of the Microsoft Windows operating system.

Noise Source	Sample Size (Byte)
GetTickCount	4
GetSystemTime	16
QueryPerformanceCounter	64

**Table 2:** Frequency of elapsed time.

Elapsed Time	Frequency
	MSB LSB
:	:
f_1_ + kd	5A561A4C
f_1_ + (k + 1)d	5A561D9A
f_1_ + (k + 2)d	5A56200D
:	5A5622E6
:	5A5625CA
:	5A562878
	5A562AE7
	5A562D66
	5A563031
	5A5632FD
	5A56357F
	5A5637DF
f_1_ + nd	5A563A7F
:	:

**Table 3:** Statistics data of Figure 2.

dt	Mean	Median	Mode
0 μs	127.43	127	209
1.0 μs	127.50	128	174
5.0 μs	127.53	128	181

**Table 4:** Statistics data of Figure 8.

dt	Mean	Median	Mode
0.1156 rad	127.56	128	210
0.2312 rad	127.59	128	45
1.1560 rad	127.62	128	132

**Table 5:** Comparison data of image quality using conventional and proposed methods.

	Conventional			Proposed		
	0 μs	1.0 μs	5.0 μs	0.1156 rad	0.2312 rad	1.1560 rad
PSNR	5.593	5.594	5.594	5.587	5.588	5.589
MSE	17,937.932	17,930.966	17,933.858	17,959.493	17,955.940	17,953.607

**Table 6:** NIST SP 800-90B test results.

	Conventional			Proposed		
	0 μs	1.0 μs	5.0 μs	0.1156 rad	0.2312 rad	1.1560 rad
min entropy	0.1089	0.1232	0.1371	7.1566	7.1578	7.1603

## Data Availability

The data presented in this study are included in this article.
